# A study of the correlation between dengue and weather in Kandy City, Sri Lanka (2003 -2012) and lessons learned

**DOI:** 10.1186/s40249-015-0075-8

**Published:** 2015-09-24

**Authors:** N D B Ehelepola, Kusalika Ariyaratne, W M N P Buddhadasa, Sunil Ratnayake, Malani Wickramasinghe

**Affiliations:** The Teaching (General) Hospital – Kandy, Kandy, Sri Lanka; Faculty of Engineering, University of Ruhuna, Hapugala, Sri Lanka

**Keywords:** Dengue, Weather, *Aedes*, Wavelet analyses, Time series, Neglected diseases, Climate change, Sri Lanka, Disease vectors

## Abstract

**Background:**

Weather variables affect dengue transmission. This study aimed to identify a dengue weather correlation pattern in Kandy, Sri Lanka, compare the results with results of similar studies, and establish ways for better control and prevention of dengue.

**Method:**

We collected data on reported dengue cases in Kandy and mid-year population data from 2003 to 2012, and calculated weekly incidences. We obtained daily weather data from two weather stations and converted it into weekly data. We studied correlation patterns between dengue incidence and weather variables using the wavelet time series analysis, and then calculated cross-correlation coefficients to find magnitudes of correlations.

**Results:**

We found a positive correlation between dengue incidence and rainfall in millimeters, the number of rainy and wet days, the minimum temperature, and the night and daytime, as well as average, humidity, mostly with a five- to seven-week lag. Additionally, we found correlations between dengue incidence and maximum and average temperatures, hours of sunshine, and wind, with longer lag periods. Dengue incidences showed a negative correlation with wind run.

**Conclusion:**

Our results showed that rainfall, temperature, humidity, hours of sunshine, and wind are correlated with local dengue incidence. We have suggested ways to improve dengue management routines and to control it in these times of global warming. We also noticed that the results of dengue weather correlation studies can vary depending on the data analysis.

**Electronic supplementary material:**

The online version of this article (doi:10.1186/s40249-015-0075-8) contains supplementary material, which is available to authorized users.

## Multilingual abstracts

Please see Additional file 1 for translations of the abstract into the six official working languages of the United Nations.

## Background

Dengue is a major public health problem in Sri Lanka. The popular local belief is that “dengue rises after a rainy spell by increasing vector population.” We believe there is a need for a better understanding of dengue weather correlation patterns after diagnosing many patients with the infection during drought periods.

We observed for dengue’s association with different weather variables. Rainfall affects reproduction of vector *Aedes* mosquitoes and therefore vector abundance [1]. However, heavy rain tends to wash out larvae from outdoor containers and reduces the lifespan of the vector [2, 3]. A certain number of rainy days is generally favorable for mosquito development [1]. Rises in temperature increase the biting rate of the vector, shorten egg to adult development time, and reduce the extrinsic incubation period of the dengue virus [2]. At 30 °C, female-to-male ratio of mosquito offspring could be 4:3 [2], and only female mosquitoes transmit dengue. Temperatures between 15–30 °C reduce adult vector mortality. The optimum temperature range for mosquito development is 25–27 °C [4]. Humidity affects flight and host-seeking behavior, and lifespan of vectors [1, 3] and vector reproduction. Humidity and wind directly affect evaporation rates of vector breeding sites [1]. Strong winds reduce mosquito density and make it difficult to find a host. However, wind also helps to extend the mosquito range [4]. On overcast days (low sunshine), vectors feed not only at the usual times of dawn and dusk, but throughout the entire day, especially if they’re indoors [5]. The effect of weather conditions and independent variables are interrelated when it comes to the outcome of dengue transmission.

Most vectors spend their entire life near the house where they were born [6]. Therefore, weather data from a distant weather station has little relevance. It is the microclimate of the locality that matters for the life cycle of the mosquito, and, correspondingly, the part of the life cycle of the virus that happens inside the mosquito’s body. We observed differences in rainfall in parts of the city and averaged data from two weather stations situated the closest to the population studied.

Published data about dengue transmission in Kandy are hard to find. We have been residents for decades and understand that dengue is a communicable disease that currently causes the greatest concern to most Kandy residents.

### Local doctors’ present (2014) knowledge about dengue weather correlation

Doctors are at the apex of both dengue preventive and curative work in Sri Lanka. In 2014 we conducted a survey among 150 doctors (of all levels of hierarchy including senior consultants, administrators, and intern medical officers of both genders who graduated from all medical faculties of the country and a couple of foreign graduates) who practice within Kandy city limits in the public and private sectors, and who contributed to dengue management in some way. We asked participants to check weather variable/s in a list and, according to their knowledge, correlate it/them with dengue incidence in Kandy, which they did. The variables were rain, temperature, humidity, wind, sunshine, and “none of them.” Each participant could select more than one variable.

The percentages of participants who selected the various weather variables were as follows: rain (89 %), temperature (32 %), sunshine (21 %), humidity (14 %), wind (7 %), rain only (41 %), and rain and temperature (15 %). Only 2 % identified all weather variables as being correlated with dengue incidence in Kandy, and 8 % indicated that none of them were correlated.

### Objective and hypotheses

Our objective was to identify weather variables and corresponding lag periods that correlated with dengue in Kandy between 2003 and 2012. Since our ultimate aim is to reduce dengue morbidity and mortality by controlling dengue better, we noted the potential ways of achieving this throughout our study.

Based on the evidence mentioned earlier, we expected to see a possible positive correlation between dengue incidence and temperature, humidity, rainfall, and number of rainy and wet days. We expected to observe a decline of dengue with heavy rain and more sunshine hours. We considered the possibility of both positive and negative correlation between dengue and wind run.

## Methods

### Study setting

To study a population affected by uniform weather, only the Kandy city area (28.53 km^2^) was selected. Estimated mid-term resident population for our study period was 114,600, with a larger floating population.

Kandy is the largest city of the central hill country (7°17′47″ N, 80°38′6″ E and 500 m above mean sea level). The climate is tropical monsoon. The mainstay of dengue prevention in Kandy and other parts of Sri Lanka is the elimination of breeding sites and aquatic forms of vector in and around dwellings. We noticed an obvious increase of this between 2003 and 2012, especially at the onset of rainy seasons and during the middle of epidemics. Fogging is done around dwellings where notified dengue patients live.

### Data collection

We obtained numbers of reported dengue cases in Kandy between January 1, 2003 and December 31, 2012 by going through the registers at the office of the city’s Medical Officer of Health (MOH). The estimated mid-year resident population for each year of the study period was obtained from the Kandy divisional secretariat office.

We obtained daily rainfall, minimum and maximum temperatures, and daytime and nighttime humidity data from the northern Katugastota and southern Gannoruwa-Peradeniya weather stations for the time period in question. In addition, we acquired the daily sunshine hours in Gannoruwa and daily wind run (wind run = wind speed _x_ duration) in Katugastota.

Then, we converted all the daily variations to weekly variations. There were a few missing data points. For example, the maximum temperature data for eight days were missing from the Katugastota weather station. If the data for one day was missing, we took the average of the available six days as the weekly data. The averages of weekly values of the two weather stations were taken as the representative values for Kandy.

### Data analysis

We used the wavelet time series analysis (wavelet analysis) to determine the correlation between dengue incidence and weather variables as the mainstay of our research.

In wavelet analysis, a suitable window is selected, which is shifted along the signal, and the spectrum is calculated for every position. Then this process is repeated many times with a slightly shorter or longer window for every new cycle. With wavelet transform, the result will be a collection of time-frequency representations of the signal with different resolutions.

Wavelet transform is an important tool as it can be used to analyze time series that contain non-stationary power at many different frequencies. By decomposing a time series into a time-frequency space, it is possible to easily determine both the dominant modes of variability and how these vary in time. Cross-wavelet transform (XWT) and wavelet coherence (WTC) can be used to examine relationships in time-frequency space between two time series. Phase angle statistics can be used to gain confidence in causal relationships and to test physical relationships between the time series.

While XWT is a common tool for analyzing localized intermittent oscillations in a time series, it is often desirable to examine two time series that may be linked in some way together. (More specifically, to examine whether regions in time-frequency space with a large common power have a consistent phase relationship and are therefore suggestive of causality between the time series.)

Continuous wavelet transform (CWT) was calculated for each weather variable under study. The idea behind the CWT is to apply the wavelet as a band pass filter to the time series. The wavelet is stretched in time by varying its scale, s, so that *η* = st, and normalizing it to have unit energy.

The CWT of a time series, X_n_, n = 1,2,…,N with uniform time step *δ*t, is defined as the convolution of X_n_ with the scaled and normalized wavelet [7].

$$ {W}_n^X(s)=\sqrt{\frac{\delta t}{s}}{\displaystyle \sum_{n\mathit{\hbox{'}}=1}^N{X}_{n\hbox{'}}{\psi}_0\left[\left(n\mathit{\hbox{'}}-n\right)\frac{\delta t}{s}\right]} $$

Wavelet power [7]:

$$ \left|{W}_n^X{(s)}^2\right| $$

A comparison of the CWT of dengue incidence with weather parameters clearly reveals common features in the wavelet power. In order to check the possibility of common power, a XWT was carried out.

The XWT finds regions in time-frequency space where the time series shows high common power. The XWT of the two time series X_n_ and Y_n_ is defined as [7]:

$$ {W}^{XY}={W}^X{W}^{Y*}, $$

where * denotes complex conjugation. Cross-wavelet power [7]: |W^XY^|

The vectors indicate the phase difference. A horizontal arrow pointing from left to right signifies “in phase” and an arrow pointing vertically upward means the second series lags the first by 90^0^.

In order to check the possibility of having a causality effect, WTC was calculated.

Wavelet coherence is defined as the square of the cross-spectrum normalized by the individual power spectra. This gives a quantity between 1 and 0, and measures the cross-correlation between two time series as a function of frequency.

If regions in time-frequency space with a large common power have a consistent phase relationship, it suggests causality between the time series [7]. The WTC is [7]:

$$ {R}_n^2(s)=\frac{{\left|S\left({s}^{-1}{W}_n^{XY}(s)\right)\right|}^2}{S\left({s}^{-1}{\left|{W}_n^X(s)\right|}^2\right)\cdot S\left({s}^{-1}{\left|{W}_n^Y(s)\right|}^2\right)}, $$

where S is a smoothing operator.

An examination the WTC and the phase arrows gives an indication that there could be a connection between dengue cases and weather parameters. In order to find the leading or lagging time, the time series were reconstructed for the period that gives the maximum power.

Since the wavelet transform is a band pass filter with a known wavelet function, it is possible to reconstruct the original time series. The reconstructed time series looks as such:

$$ {x}_n=\frac{\delta j\delta t\raisebox{1ex}{$1$}\!\left/ \!\raisebox{-1ex}{$2$}\right.}{C_{\delta }{\psi}_0(0)}{\displaystyle {\sum}_{j=0}^J\frac{\Re \left\{{W}_n\left({s}_j\right)\right\}}{s_j^{\raisebox{1ex}{$1$}\!\left/ \!\raisebox{-1ex}{$2$}\right.}}}, $$

where, ψ_0_(0) removes the energy scaling and $$ {s}_j^{\raisebox{1ex}{$1$}\!\left/ \!\raisebox{-1ex}{$2$}\right.} $$ converts the wavelet transform to an energy density. The factor *C*_*δ*_ is a constant for each wavelet function.

Considering the above equation and by summing over a subset of the scales, it is possible to construct a wavelet-filtered time series. In the present study, the period that gives the highest coherence among the dengue cases and each of the weather parameters was identified. The wavelet-filtered time series for the period were reconstructed and the lagging times were estimated. Our CWT can be considered as a pre-whitening multi-scale matched filter [8].

Wavelet analysis was done using the MATLAB R2013a software (MATLAB Corporation, USA). The wavelet results do not show magnitudes of correlation. To get an idea about magnitudes, mainly to compare various rain parameters, cross-correlation coefficients between dengue incidence and weather variables were calculated using SPSS Statistics 20 software (IBM Corporation, USA). We calculated cross-correlations for minus 20 to plus 20 weeks. We selected a 20-weeks limit because realistic lag periods are likely to be less than that, as described in the discussion.

We were interested in comparing our results with results of a recent study done in Indonesia [4], as it was done in a place with similar (albeit a southern) latitude and also studied several weather variables. We observed that this study determined dengue weather variable correlations using two-tailed Spearman’s correlation coefficient (without lag periods). We also calculated the same for our data for comparison. The results were added to Table 1.

**Table 1:** Summary of wavelet analysis, cross-correlation coefficient analysis, and Spearman’s rho results

Parameters	Wavelet analysis (MATLAB)	SPSS	
	Sign of correlation and (lagging period in weeks)	Cross-correlation coefficient	Spearman’s rho
Dengue incidence vs. rainfall (in mm)	Positive (7 weeks)	−0.143 in 18 weeks (0.069 in 7 weeks, but below upper confidence level)	−0.095; correlation is significant at the 0.05 level (two-tailed)
Dengue incidence vs. rainy days (days with rainfall >0.1 mm)*	No wavelet coherence	0.146 in 7 weeks	−0.058
Dengue incidence vs. rainy days (days with rainfall >0.3 mm)*	Positive (7 weeks)	0.144 in 7 weeks	−0.70
Dengue incidence vs. wet days (days with rainfall >1 mm)*	Positive (7 weeks)	0.137 in 7 weeks	−0.61
Dengue incidence vs. wet days (rainfall >3 mm)*	Positive (7 weeks)	0.136 in 7 weeks	−0.074
Dengue incidence vs. days with rainfall >20 mm	Positive (7 weeks)	−0.133 in 18 weeks (0.049 in 5 weeks, but below upper confidence level)	−0.116; correlation is significant at the 0.01 level (two-tailed)
Dengue incidence vs. maximum temperature	Positive (12 weeks)	0.202 in 14-week lag; 0.139 in 12-week lag	−0.143; correlation is significant at the 0.01 level (two-tailed)
Dengue incidence vs. minimum temperature	Positive (6 weeks)	0.166 in 7 weeks	−0.035
Dengue incidence vs. average temperature	Positive (11 weeks)	0.214 in 14 weeks	−0.169; correlation is significant at the 0.01 level (two-tailed)
Dengue incidence vs. daytime humidity	Positive (5 weeks)	0.158 in 7 weeks	−0.038
Dengue incidence vs. nighttime humidity	Positive (7 weeks)	0.137 in 7 weeks	−0.135; correlation is significant at the 0.01 level (two-tailed)
Dengue incidence vs. average humidity	Positive (5 weeks)	0.161 in 7 weeks (0.131 in 5 weeks)	−0.066
Dengue incidence vs. wind run	Negative (9 weeks)	0.181 in 20 weeks	0.150; correlation is significant at the 0.01 level (two-tailed)
		-0.042 in 7 weeks	
Dengue incidence vs. sunshine hours	Positive (15 weeks)	0.130 in 18 weeks (0.113 in 15 weeks; 0.125 in 6 weeks)	0.014

*The Sri Lankan department of meteorology defines a rainy day as a day with rainfall >0.3 mm (in some countries, it is >0.1 mm), and a wet day as a day with rainfall >1 mm. The Sri Lankan department of agriculture defines wet day as a day >3 mm rain. We have done calculations for all these definitions

### Ethics statement

We collected only the number of reported (notified) dengue cases and didn’t collect any information about patients’ identities. The study was approved by the Health Ministry of Sri Lanka (ETR/E/MC/RP/350/2012).

## Results

**Fig. 1:**
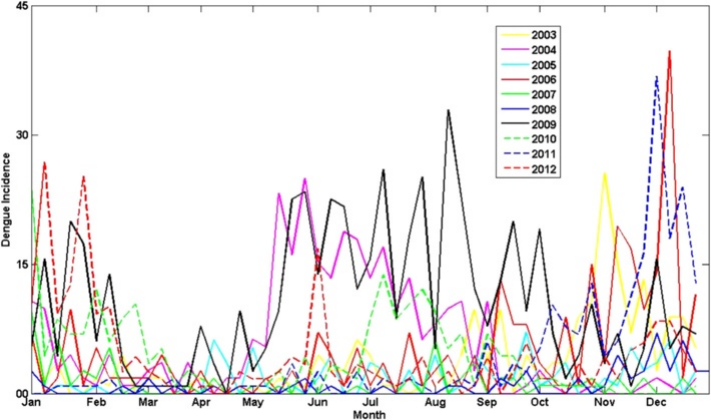
Variations of weekly dengue incidence (per 100,000 population) during the course of 52 weeks of each year, 2003–2012

**Fig. 2:**
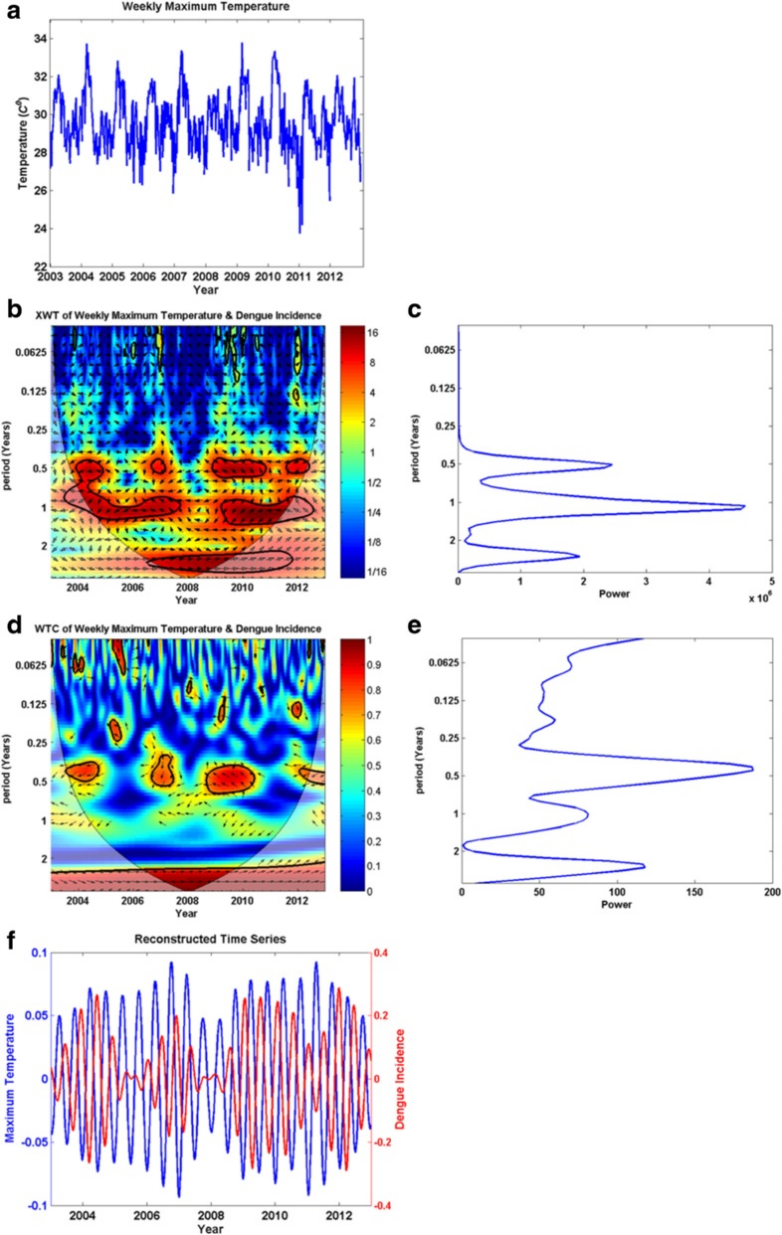
Wavelet analysis results for the maximum temperature versus dengue incidence time series as a sample (2**a**–2**f**). **a** Weekly average maximum temperature (x-axis: year, y-axis: weekly average maximum temperature); **b**) Cross-wavelet transform (XWT) (x-axis: year, y-axis: period in years); **c**) XWT power for each period (x-axis: power, y-axis: period in years); **d**) Wavelet coherence (WTC) (x-axis: year, y-axis: period in years); **e**) WTC power for each period (x-axis: power, y-axis: period in years); **f**) The time series relevant to maximum wavelet coherence is reconstructed, and shown in this figure. In Figures 2**b** and **d**, there are color codes on the right side of the main figure. These indicate the magnitude of XWT and WTC; dark blue and dark red indicate lowest and highest magnitudes respectively. The thin parabolic black line demarcates the cone of influence

As shown in Fig. 1, which illustrates how weekly dengue incidences have changed during the course of each year between 2003 and 2012, dengue incidence in Kandy was usually high from October to February. This rise occurred following second intermonsoon rains and during northeast monsoon seasons. In 2004, 2009, and 2010, dengue peaked between May and September during the southwest monsoon season. Figure 2 shows a sample of the wavelet analysis results.

### Results of the wavelet and correlation coefficient analyses

The wavelet analysis showed a cyclic pattern of dengue incidence and variations in weather variables in six-month, one-year, and two-and-half-year cycles..

Wavelet analysis showed that maxima in all the considered weather variables increase dengue incidence with a lag period, except for wind run. Maxima of wind run were followed by a decline in dengue incidence.

From the cross-correlations results, we selected the value of the highest magnitude above confidence intervals for each weather variable.

Table 1 summarizes the results of the wavelet, cross-correlation coefficient, and the Spearman’s rho analyses.

Correlation patterns and lag periods are generally (but not always) in agreement with wavelet and cross-correlation coefficient results. In the discussion, we examine which result is more realistic considering the biology of the vector and virus when results from these two methods differ. The change of dengue incidence happens after a seven-week lag period for the majority of weather variables. Wavelet analysis is a better method to study non-stationary correlations such as that between dengue and weather variables. Therefore, we decided to give more emphasis to the specific details of the wavelet results. Spearman’s rho results contradicted the results of the other two methods. We discuss this and the correlation patterns of individual weather variables in the discussion section.

During our study period, the average mean daily temperature in Kandy was estimated to be 25.1 °C, the average daytime relative humidity was estimated to be 73 %, and the average nighttime relative humidity was estimated to be 88 %. The average daily wind run was 3.5 km. There were 5.9 hours of daily sunshine on average and the average annual rainfall was 1954.2 mm. The average annual dengue incidence was 219 per 100,000 population.

Considering the temperatures in which dengue was shown to rise in Thailand (1983–2001) and Singapore (2000–2012) [9, 10], we attempted to find a correlation between dengue incidence and the number of days per week with a mean temperature >27.8 °C, but abandoned this idea as we only had 18 such days in our 10-year study period.

## Discussion

Many studies have been done in various tropical and subtropical regions on dengue weather correlation. Most of them studied the effects of rainfall, temperature, and humidity. Of these, the majority found that dengue was correlated with these weather parameters to variable degrees, with lag periods varying from a few weeks to a few months. A great majority of them studied larger areas and populations, but did not analyze weather variables in detail, as we did in this study.

Our Spearman’s rho results contradicted the results of the other two methods. We respect the efforts of past researchers who had faith in Spearman’s rho without lag periods [4], but considering the reasons outlined below, we believed that this method was unsuitable for our study and hence didn’t want to further interpret Spearman’s rho results. Spearman’s rho is good for measuring linear and stationery relationships, but dengue weather correlation is not stationary or linear. To use this method, the relationship should be monotonic. But when we consider rain versus dengue incidence, regular rain favors an increase in dengue, but heavy rain may not. Further, the method does not automatically calculate a lag period. When we consider the biology of the vector and the dengue virus, it is clear that there should be lag periods.

However, we learned an important lesson from the Spearman’s rho calculations that we would like to share. We have highlighted this as an example of how the selection of statistical methods drastically alters study results. Statistics is not the forte of most healthcare workers. Unless the reader has a good knowledge of statistics and gives serious attention to the analysis part of the study, he/she may get an inaccurate picture of similar studies, especially when comparing results of two studies. If someone can do a study and find analytical methods that can be recommended as “standard” and that give comparable results in dengue weather correlation studies, future researchers shall stick to those methods, as the results will be more useful. This issue may be relevant to other research fields as well.

### Correlation patterns of individual weather variables

Our wavelet analysis shows that all studied weather variables except the wind run were positively correlated with dengue incidence in Kandy. The wind run showed a negative correlation with dengue, as similar to other studies done in Vietnam (1998–2009) [11] and Barbados (1995–2000) [12]. Wind may increase the range of vector mosquitoes [4], which may consequently increase the chance of more humans being infected. This effect appears to get negated by other effects of wind, as discussed in the background section. As Kandy has a high population density, we can assume mosquitoes can find a human to feed on without the wind having to disperse them far. The cross-correlation method gave a 20-week lag and a positive correlation. Even if we accept the positive correlation considering the wind’s contribution in increasing the range of vector mosquitoes, the 20-week lag period is less likely to occur than a nine-week lag (likely durations of lag periods for our study are explained later). Therefore, wavelet results appear to be more realistic.

We can speculate the possibility of the wind dispersing vector mosquitoes (dengue-infected) and reducing dengue incidence first, then later those vectors and their offspring (dengue virus is vertically transmitted in *Aedes* mosquitoes) causing dengue in new localities and contributing to a rise in the dengue incidence. But more studies are needed to verify this hypothesis.

We also found a positive correlation between dengue incidence and hours of sunshine. On overcast days, *Aedes* mosquitoes bite throughout the day [5]; these days are usually also humid and thus favor dengue transmission. However, we observed that other stronger effect/s override this. One possible explanation is that during sunny days, more people are outdoors, especially at dawn and dusk, exposing them to *Aedes* mosquito bites. Also, outdoor mosquito flight is not hindered by rain. Another possible explanation is that more hours of sunshine mean more solar radiation on Earth, which increases the temperature, especially the daytime (maximum) temperature, and this in turn can contribute to the rise in dengue. To check this hypothesis, we calculated cross-correlation coefficients between sunshine hours and maximum and mean temperatures for our study period; they were 0.725 and 0.325 respectively with no lag. These correlated well, indirectly indicating that the effect of sunshine hours on dengue incidence was likely to be via the rise in temperature.

We observed a negative correlation between the hours of sunshine and dengue incidence in a six-week lag; the magnitude is a little lower than in the 20-week lag. A six-week lag is more likely to be realistic than a 18–20-week lag. But as our mainstay wavelet results also gave positive correlations, we took it as positive. Future studies may verify the exact nature of hours of sunshine and dengue correlation. But all our analysis results confirmed that they are correlated.

We also observed that past researchers who have studied dengue weather correlation have not evaluated the correlation between weather variables. *Aedes* mosquitoes are rapidly spreading further from the equator to places where sunshine hours and temperatures show wide fluctuations with seasons [13]. Considering this, we believe the effect of sunshine hours on dengue incidence deserves further study.

The negative correlation we observed between rainfall in millimeters and dengue incidence in cross-correlation studies is unrealistic when we consider the biology of the vector, and a 18-week lag is also less likely than a seven-week lag. Rainfall affects dengue mainly by increasing breeding sites for the vector. In places where people store water in open containers during dry seasons, a negative correlation may occur. But in Kandy city, a great majority of the population including the poor has direct or indirect access to pipe-borne water throughout the year. Thus, we considered only wavelet results for this weather parameter. A study done in an area adjacent to Kandy in 2007/8 [14], which showed that the number of *Aedes* eggs (indicator of vector abundance) positively correlated with rainfall further supports this decision.

We observed that both >1 mm and >3 mm rainfall wet days and >0.3 mm rainy days showed correlation with dengue in wavelet studies and similar magnitude cross-correlations as rainfall in mm (rain parameter used by most past researchers). Therefore, the number of rainy days (rainfall >0.3 mm) and wet days are equally suited to understanding the correlation between dengue incidence and rainfall in Kandy. This may also be applicable elsewhere.

We expected to see a possible negative correlation between dengue incidence and number of days with >20 mm rainfall. However, these were positively correlated in wavelet studies, and showed lesser magnitude but negative correlation compared to other rain parameters in cross-correlation coefficient calculations. A possible explanation is that heavy rainfall creates more breeding sites and deeper levels of water allowing mosquitoes to complete their aquatic life cycles. This may compensate, to a certain extent, to the effect of some larvae getting washed off.

We also found that minimum, mean, and maximum temperatures are correlated with dengue incidence, as in the majority of studies done in other tropical and subtropical regions [1–3, 12, 14]. A comprehensive study conducted in Thailand between 1983 and 2001 shows that 80 % of severe dengue cases occurred when the mean temperature was 27–29.5 °C and the mean humidity was >75 % [10]. Temperature is the most important weather variable affecting dengue transmission according to studies done in Thailand (1983–2001) and Singapore (2000–2007) [9, 15]. A study done in Karachi, Pakistan, between 2005 and 2009 showed that 77 % of dengue fever cases occurred when humidity was around 80 % and the temperature was 30–32 °C [16]. In comparison, the mean temperature and humidity of Kandy during our study period were 25.1 °C and 80 % respectively, and the correlation coefficient between dengue incidence and number of days per week with mean humidity >80 % was 0.136 with a seven-week lag.

The nighttime (maximum), daytime (minimum), and average weekly humidity for our study period were 88 %, 72 %, and 80 % respectively. The abovementioned Thailand study [9] showed that between 1983 and 2001, dengue incidence maximized above 88 % maximum humidity, 55 % minimum humidity, and 75 % mean humidity. Accordingly, it appeared that humidity in Kandy was very favorable for dengue transmission.

### Non-weather factors affecting dengue incidence

Reliability of results of dengue weather correlation studies depends on the accuracy of the dengue and weather data. Factors other than weather affect dengue. These include herd immunity of population, introduction of new dengue virus genotypes to the population, urbanization, effectiveness of preventive programs, population movement, and socioeconomic factors such as quality of housing and garbage disposal, dengue awareness, and people’s attitudes [3, 10]. A detailed systematic analysis of all these factors is beyond the scope of this study. There is also a lack of data regarding many of these factors. However, we would like to mention some key points from the available information that we believe will help construct a better picture of the dengue status in Kandy. We didn’t find any information about herd immunity/seroprevalence for dengue in the Kandy population. However, a study done in 2008 in Colombo (115 km away) showed approximately 30 primary dengue infections among children <12 years in the community for every single case that was notified [17]. We think the Kandy situation is not dissimilar from this.

The rise in dengue in the later months of 2003 and 2004, late 2006, 2009, 2010, late 2011, and early 2012 all correspond to national-level dengue epidemics [18]. The 2002–2004 dengue epidemic of Sri Lanka was attributed to the introduction of a new clade of dengue virus serotype 3, and the 2009 epidemic was attributed to an introduction of a new genotype of dengue virus serotype 1, with the effect of that continuing in the following years [19]. We believe dengue epidemics that affected Kandy during those years were influenced by the introduction of these new virus genotypes.

In addition to the resident population, a larger population commutes to Kandy daily. According to the 2012 budget report of the Kandy municipal council, this floating population is estimated to be 125,000. A 2007 study showed that approximately 50,000 vehicles enter and exit the city in an average working day [20]. A few of these may be bringing in/taking out dengue-virus-infected vectors and patients with dengue viremia. Kandy is one of the most popular destinations in Sri Lanka for local as well as foreign tourists and pilgrims. Local visitors arrive from all provinces of the country. Precise data about numbers and origin of foreign visitors to Kandy are not available, but according to a survey done in 2011, 63 % of tourists who came to Sri Lanka visited Kandy. According to the 2012 annual statistics report of the Sri Lanka tourism development authority, the largest number of tourists to Sri Lanka originated from India. Other dengue endemic countries such as the Maldives, Singapore, and Malaysia also contributed a good number of tourists. On average, a tourist spends 10 nights in the country.

Colombo and the surrounding areas are considered Sri Lanka’s dengue “hotspot.” As practicing clinicians, we have seen Kandy residents working in Colombo get infected there and treated here.

Mass population movements are also attributing to dengue epidemics [21]. In mid-April and July/August, mass population movements occur in Kandy due to national festivals.

We didn’t find any published information about other factors that affect the local dengue incidence. However as citizens of the city, we have observed that Kandy has become more urban, garbage disposal has improved, and there has been an intensification of *Aedes* population control efforts and dengue awareness programs. The annual dengue incidence of Kandy was higher than the national incidence of Sri Lanka and most other endemic countries during our study period.

### Estimation of realistic lag periods

We didn’t find any published studies about realistic lag periods, or even about the average life span and survival rates of *Aedes* mosquitoes in Sri Lanka. Therefore, we decided to make an estimate based on the available information. Singapore and US government agencies respectively give the average life span of *Aedes* mosquitoes in nature as two and three weeks [22, 23]. There are also reports of longer life spans of *Aedes* reared in laboratories, but we did not consider these.

First, we would like to describe the regular dengue transmission pattern. It takes one to two weeks for an *Aedes* egg to develop into an adult. Soon afteremerging pupae, *Aedes* mosquitoes do not suck on humans or lay eggs. After a female *Aedes* mosquito sucks blood from a patient with dengue virus, it takes eight to 12 days for the virus to multiply and reach the salivary gland of the mosquito, which enables it to infect a human. This time period is called the extrinsic incubation period. Once a healthy person gets the virus from a mosquito, it takes another three to 14 days, most commonly four to seven days, for symptoms to appear. This period is called the intrinsic incubation period. Our experience is that patients generally come to a primary care doctor on the second or third day, or later after an onset of the symptoms. After the third day of clinical suspicion, full blood count (complete blood count) is done and if platelet count drops below 100,000 mm^3^ (especially with a high hematocrit), patients are directed for inward care. After 2012, dengue NS1 test availability in the private sector made early-confirmed diagnosis of some cases possible.

As explained earlier, weather variables affect *Aedes* mosquito reproduction. If we consider an *Aedes* egg laid today, this week’s weather will affect its life cycle. We have to consider that it will take two to three weeks once a healthy person gets the dengue virus into their body to get notified into the registers of the Kandy MOH. Hence, five to eight weeks is the likely lag period in our study. However, if we consider an adult vector, this week’s weather is likely to affect not only its life cycle but its reproduction, to a certain extent, as well. Considering the next generation, an eight to 11-week lag period is likely. Therefore, considering all of the above, the likely lag period for Kandy is five to 11 weeks.

Sometimes if the water collections dry out, *Aedes* eggs can survive longer and hatch again when it rains. Singapore’s *Aedes* mosquito control mechanisms may be more efficient than that in Kandy, consequently making vector lifespan here a little longer. The lag periods that we determined by our analyses, especially by wavelet analysis, are generally compatible with the biology of the vector and the dengue virus according to these estimates. To get a better idea of the realistic lag periods for Kandy, a dedicated study needs to be done.

### Comparing results of similar studies from the region

Our literature review found three recent studies done in Sri Lanka, however, none of them were from the central hill country. Table 2 summarizes these studies.

**Table 2:** Summary of recent similar published studies done in Sri Lanka

Place	Year published	Study period	Dengue notified/seropositive	Weather variables studied	Correlation identified
Colombo, Ratnapura, and Anuradhapura districts [21]	2013	2005–2011	Notified dengue cases	Tmax, rainfall	Tmax. and rainfall did not affect dengue incidence (but there was a mild correlation between dengue and rainfall in two cities).
Gampaha (it was one of the six Asian urban areas studied) [24]	2012	2006–2009	Seropositive dengue	Rainfall	A positive correlation was observed between the number of dengue cases and rainfall.
Western province of Sri Lanka (Colombo, Kalutara, and Gampaha districts combined) [25]	2009	2000–2004	Notified dengue cases	Rainfall	Dengue incidence was relatively low during heavy rainfall and increased when rainfall started to decrease, showing a 3–4-week lag. Dengue was strongly correlated with rain in most of the studied towns.
					(No significant variations of temperature and humidity in were found, so they were not considered.)

*Tmax* = maximum temperature

The results of the first and third studies [21, 24], as shown in Table 2, done in Colombo district in different time periods differ although they used data from the same source. This indicates a need for more studies to be done in order to understand dengue weather correlation in Sri Lanka. The inconsistency may be due to differences in data analysis methods as discussed above. This again illustrates the value of identifying data analysis methods that give comparable results. Similarly, results of two studies done in southern Thailand during different time periods (1993–2002 and 1978–1997) also differ [1]. Two studies done in Guangzhou, China in different time periods (2000–2006 and 2007–2012) show negative and positive correlations between dengue incidence and wind velocity [26, 27]. Therefore, variations in correlation patterns between weather variables and dengue incidence even in the same locality over time is another possibility we have to think of.

In an area adjacent to the southern border of Kandy, a study done in 2007/8 showed no positive relationship between the abundance of *Aedes* eggs, and larval density indices (that gives an idea of vector abundance) and the number of dengue cases. A positive relationship between the number of *Aedes* eggs and rainfall and humidity was found, but not with temperature [14]. This result contrasts with the results of a study done in Thailand (1990–1993) [28], where the temperature correlated with fluctuations in dengue vector abundance but not with rainfall. This indicates the role of the mechanism/s other than vector abundance in causing the rise in dengue in Kandy; these also appear to be more prominent than the effect of rainfall increasing vector abundance. (But do note that the duration of the above study [14] was only one year.) We believe the likely mechanisms are a rise in temperature accelerating the life cycle of the dengue virus inside the vector and the biting frequency of the vector [2, 14]. We have observed obvious intensification of vector population control methods from 2003 but dengue epidemics have become more frequent. Hence, it is rational to give more attention to other methods in addition to existing vector population control methods for dengue control.

We noticed that there have been more studies on this topic published from neighboring South Asian countries after the start of our study in 2012. We include a summary of these in Table 3. (Note that the last item was published before the start of our study).

**Table 3:** Summary of findings of recent similar studies done in other South Asian countries

Place	Year published	Study period	Dengue notified/seropositive	Weather variables studied	Correlation identified
1. Dhaka, Bangladesh [29]	2014	2000–2010	Notified dengue cases	Tmax, Tmin, rainfall, R.H.	Monthly temperature and humidity were significantly associated with monthly dengue incidence with highest lag effect of four months.
2. Tamil Nadu, (South) India [30]	2013	2000–2008	Notified dengue cases	Monthly mean Tmax and Tmin, rainfall	Rainfall and temperature influence dengue incidence. Climatic variance in high incidence and low incidence years does not show any difference. Both rain and drought are conducive to surges of dengue.
3. Dhaka, Bangladesh [31]	2012	2000–2008	Notified dengue cases	Rainfall, Tmax, R.H.	Rainfall, Tmax, and R.H. significantly correlated with monthly reported dengue cases.
4. Lahore (North), Pakistan [32]	2012	2007–2011	Seropositive dengue	Tmin, Tmean, rainfall, R.H.	Tmin, Tmean, R.H., and rainfall all had significant positive correlation with dengue with four-, six-, and eight-week lags. Strongest correlation with rainfall was with an eight-week lag. Tmax had no significant correlation.
5. Lucknow, (North) India [33]	2012	2008–2010	Seropositive dengue (hospital-based study)	Tmin, Tmax. R.H Rainfall	No statically significant correlation between dengue and weather variables.
6. Manipur, (North East) India [34]	2012	2007–2008	Seropositive dengue	Tmin, Tmax, morning and afternoon R.H., rainfall	Dengue has not been reported in Manipur until the 2007 outbreak. Changes in the weather were studied between 2005 and 2008, compared to 2000–2004. A significant increase in Tmin, rise of morning R.H, a decrease of afternoon RH, and a decrease of rainfall was found in the 2005–2008 period.
7. Karachi, (South) Pakistan [16]	2011	2005–2009	Notified dengue	Rainfall, R.H., temperature	Ambient temperature, humidity and post-monsoon rain results increased mosquito activity with consequential higher incidence of dengue.

*Tmax* = maximum temperature; *Tmin* = minimum temperature; *Tmean* = mean temperature; *R.H*. = relative humidity

We studied weekly dengue incidence in terms of weather variables (rather than monthly dengue incidence), which produces a more precise idea about their correlation. We also noticed a lack of similar studies done in the African continent. Table 4 outlines recent reviews done on dengue weather correlation.

**Table 4:** Recent reviews on dengue weather correlation

Year Published	Short description	Reference
2013	Results of a dengue weather correlation study in Malaysia. Also describes results of similar studies.	Cheong YL, Burkart K, Leitao PJ, Lakes T. Assessing weather effects on dengue disease in Malaysia. International Journal of Environmental Research and Public Health 2013; 10: 6319–6334.
2013	Describes climate change and mosquito-borne diseases in China, and includes an informative table about dengue.	Bai L, Morton LC, Liu Q. Climate change and mosquito-borne diseases in China: a review. Global Health 2013; 9: 1–22.
2013	Summarizes findings of 31 studies done in various parts of Brazil and concludes that dengue is strongly related to meteorological variables.	Viana DV, Ignotti E. The occurrence of dengue and weather changes in Brazil: a systematic review. Revista Brasileira de Epidemiologia 2013; 16: 240–256.
2012	Summarizes findings of 10 long-term studies from the Asia-Pacific region and America about ENSO and dengue correlation.	Thai K TD, Anders KL. The role of climate variability and change in the transmission dynamics and geographic distribution of dengue. Experimental Biology and Medicine 2011; 236: 944–954.
2011	Summarizes findings of 22 studies from the Asia-Pacific region about dengue weather correlation.	Banu S, Hu W, Hurst C, Tong S. Dengue transmission in the Asia‐Pacific region: impact of climate change and socio‐environmental factors. Tropical Medicine & International Health 2011; 16: 598–607.

### Lessons learned

Previous authors have not proposed any means of mitigating effects of higher temperatures on dengue incidence. We believe mitigating effects of increasing biting frequency by vectors more likely to have dengue virus in their saliva is an important way to control the virus spreading. We recommend educating people, especially in high-risk areas, to apply mosquito repellent to exposed body parts when going outdoors on hot days, especially during dawn and dusk, as a habit (similar to applying cosmetics before going out). We also encourage all patients in dengue prevalent areas with fever, headache, and joint and muscle aches during the first few days (until diagnosed) to apply repellents in the morning and evening to minimize potentially spreading the disease. Also it is advisable to use repellent impregnated nets on windows and grills, and fogging when appropriate. These measures may help to mitigate effects of low wind as well. Wearing garments covering as much of the body as possible is another option, however, this interferes with activity (sports, working in paddy fields, etc.) and can be uncomfortable in warm and humid tropical climates. Pilot studies are needed to confirm relative efficacies of these measures.

Studies done in Thailand (1983–2001), Singapore (2000–2007), and an area of Pakistan (2005–2009) have revealed that most dengue transmission occurs at certain temperature ranges that vary from country to country [9, 15, 16]. Finding this range for various districts of Sri Lanka and then warning the public in advance (using mass media) to practice preventive measures when the temperature is in that range is one solution.

With the backdrop of global warming, considering the enormous and fast-growing population at risk, and as so many studies from different countries indicate dengue temperature correlation, establishing ways to counter the effect of rising temperatures deserves priority.

Making dengue NS1 antigen test and IgM antibody test freely available at Sri Lankan state hospitals will make dengue databases more accurate. There are many additional benefits. This test availability will help early-confirmed diagnosis of dengue at state hospitals and will therefore assist to better manage dengue patients, as well as providing more efficient and cost-effective preventive work.

When a lot of cases with many false positives get notified during a dengue epidemic, quality of preventive healthcare work can get compromised at a time when it is most crucial. Additionally, it is costly to send a preventive health team to investigate each false positive case and do fogging. If a false positive case is in fact another important infection, the gravity of the problem will be even greater because preventive work of that infection also gets compromised. For example, Kandy’s MOH personally communicated during the 2009 epidemic that some reported dengue cases were further investigated and later serologically found to be influenza H1N1. There was a pandemic of influenza H1N1 in 2009.

Enabling outpatient department (OPD) doctors to order dengue NS1 antigen tests from hospital laboratories can help in early confidant diagnosis. Then, the notification process can come from the OPD itself using electronic means for quicker notification. If this happens, someone can research daily dengue cases in terms of daily weather variables studies and get a more precise idea of the correlation patterns involved. Taking action in the early stages of epidemics helps to contain them and reduce morbidity and mortality. Our hospital has already taken certain steps towards a quicker notification process.

The dengue NS1 antigen test is very specific and can diagnose dengue on the very first day a patient comes to see a doctor [35, 36]. Sometimes, it can help to refine patient management decisions. For example, leptospirosis is another emerging infection that comes to the differential diagnosis of a considerable number of our dengue patients. We start antibiotics in such patients on admission in addition to the usual dengue management. Availability of NS1 antigen test will help to avoid this in confirmed dengue patients. Some patients with fever present with nonspecific symptoms and signs later get diagnosed as dengue patients. Some doctors prescribe an antipyretic/analgesic (paracetamol) only *pro re nata* for patients during the first couple of days until a diagnosis is reached. This is done to find the fever pattern that is sometimes helpful for diagnosis. Fever and pain of dengue are serious and called “break bone fever.” An additional benefit of the NS1 antigen test is, if positive, doctors can start a regular dose of paracetamol early, which will reduce the patient’s pain and fever.

The NS1 antigen test will help doctors diagnose dengue more confidently and early so non-dengue patients with similar symptoms can be sent home early and reduce overcrowding in hospitals that leads to numerous problems. This is especially important during epidemic times, with dengue epidemics becoming more frequent in the recent past. The dengue NS1 antigen test has some weaknesses, such as the cost, risk of giving false positives with Japanese encephalitis virus (another rarer mosquito-borne infection in Sri Lanka), and that it’s not very useful a few days after the onset of fever. However, we still believe the benefits outweigh the weaknesses. For accurate diagnosis of patients who come to hospitals later, the availability of IgM test will be helpful.

Using GIS (geographic information system) technology to store data about dengue and other diseases will be a useful investment. We think these observations are applicable to other parts of Sri Lanka and other developing countries to a certain extent as well.

For efficient preventive work, notification of all diagnosed patients as far as possible is important. One of the byproducts is that dengue databases will be more accurate. During our study period, a great majority of the reported dengue cases were from the three government teaching hospitals in the city and private sector contribution was minimal. For example, in 2012, all private institutions contributed less than 2 % of the notified dengue patients. After talking with some knowledgeable people, we think the most likely reason is that some people do not like public health inspectors coming and inspecting their homes and neighborhoods for mosquito-breeding sites. (A few years ago, the government introduced fines for owners of properties with mosquito breeding sites. But this hesitancy was there even before). Furthermore, some believe reporting makes them unpopular. To our best knowledge, this is true for many other private hospitals in the country.

For the greater benefit of society, we have to find means of getting more diagnosed cases notified from the private sector without harming its interests, and to regularly remind state doctors about the importance of notification.

Dengue incidence is very high in Sri Lanka and almost every doctor is alert about it. However in our survey only 2 % of local doctors indicated all weather variables of our study are correlated with local dengue. Doctors are the decision-makers in dengue preventive and curative work. That indicates the need to improve local doctors’ awareness of the topic. We therefore think our study contributes to the advancement of existing local knowledge on dengue weather correlation and, consequently, to an improvement of local dengue control.

## Conclusion

Our study showed correlations between dengue incidence and rain, temperature, humidity, hours of sunshine, and wind in Kandy, with lag periods of several weeks.The effect of sunshine hours on the local dengue incidence appears to be via the rise of temperature. The number of rainy and wet days may be equally apt to study the rain’s relationship with dengue incidence. Our findings were broadly similar to the results of many published studies conducted in various tropical and subtropical countries.

We recommend that while maintaining existing dengue prevention programs throughout the year, more emphasis should be placed on topical application of mosquito repellents to mitigate the effects of temperature on dengue incidence. We believe this has global application value, especially with the backdrop of global warming.

Sometimes, results of dengue weather correlation studies vary depending on the analytical method used. Therefore, we advise comparing the results of various studies with caution.

Availability of the dengue NS1 antigen test for early confident diagnosis at state hospitals and finding methods for a larger number of diagnosed dengue cases to get reported from the private sector will improve accuracy of dengue databases. These two aspects and the use of modern information technology in dengue preventive work, communications, and data storage will improve management of the dengue situation. We believe these suggestions are applicable elsewhere as well.

## Additional file

Additional file 1:
**Multilingual abstracts in the six official working languages of the United Nations.** (PDF 346 kb)

## Abbreviations

CWT: Continuous wavelet transform
MOH: Medical Officer of Health
OPD: Outpatient department
WTC: Wavelet coherence
XWT: Cross-wavelet transform
