# Supplementary material for: A study of the correlation between dengue and weather in Kandy City, Sri Lanka (2003 -2012) and lessons learned
Source: Infect Dis Poverty. 2015 Sep 24;4:42. doi: 10.1186/s40249-015-0075-8 (PMC4581090; doi:10.1186/s40249-015-0075-8)
Supplement: Additional file 1: — Multilingual abstracts in the six official working languages of the United Nations. (PDF 346 kb) [file 40249_2015_75_MOESM1_ESM.pdf]

Translation of the abstract into the six official working languages of the United Nations

دراسة العلاقة بين حمى الضنك والطقس في مدينة كاندي بسريلانكا فى الفترة ما بين عامى 2003 و 2012ن.د.ب. إليبولا، كوساليكا أريارانتى،

و.م.ن.ب. بوداسا، سونيل راتناياك و مالانى ويكراماسنجي

الملخص

الخلفية العلمية: نظرا لتأثير متغيرات الطقس على انتقال حمى الضنك فقد هدفت الدراسة الحالية إلى التعرف على نمط الإرتباط بين الطقس وحمى الضنك في مدينة كاندي بسريلانكا ومقارنة النتائج مع نتائج دراسات مماثلة وإنشاء

طرق تحسين المراقبة والوقاية من حمى الضنك.

طريقة البحث: تم جمع البيانات عن حالات حمى الضنك التى تم الإبلاغ عنها في كاندي وكذلك البيانات السكانية فى الفترة ما بين عام 2003 الى عام 2012، وتسجيل حوادث الإصابة الأسبوعية المحسوبة وبيانات الطقس اليومية التى تم الحصول عليها من اثنتين من محطات الأرصاد الجوية بعد تحويلها إلى بيانات أسبوعية، تم إستخدام البيانات السابقة فى دراسة أنماط الإرتباط بين حالات حمى الضنك ومتغيرات الطقس وذلك بإستخدام تحليل السلاسل الزمنية المويجى ومن ثم حساب معاملات الإرتباط المتداخلة للعثور على مقادير الإرتباطات.

النتائج: وُجد أن هناك علاقة طردية بين حدوث حمى الضنك وكلا من هطول الأمطار بالملليمتر، وعدد الأيام الممطرة والرطبة، والحد الأدنى لدرجة الحرارة، والليل والنهار، وكذلك متوسط الرطوبة، ومعظمها مع فارق خمسة إلى سبعة أسابيع. بالإضافة إلى ذلك أظهرت الدراسة وجود علاقة طردية بين حالات حمى الضنك ودرجات الحرارة القصوى ومتوسط عدد ساعات سطوع الشمس، والرياح، مع فترات تأخير طويلة بينما كانت العلاقة بين حالات حمى الضنك وجريان الرياح علاقة سلبية.

الإستنتاج: بينت النتائج الحالية أن هطول الأمطار ودرجة الحرارة والرطوبة، وساعات سطوع الشمس، والرياح ترتبط مع حدوث حمى الضنك محليا فى مدينة كاندى وبالتالى تم إقتراح طرق لتحسين الإجراءات الروتينية لإدارة حمى الضنك والسيطرة عليها في ظل ظاهرة الاحتباس الحراري الحالية، أيضا وُجد أن نتائج دراسات ارتباط الطقس بحمى الضنك يمكن أن تختلف تبعا لكيفية تحليل البيانات.

Translated from English version into Arabic by Mohamed R. Habib, through

CHINESE CENTER FOR DISEASE CONTROL AND PREVENTION

NATIONAL INSTITUTE OF PARASITIC DISEASES

斯里兰卡康提市气象和登革热的相关性研究

N. D. B. Ehelepola, Kusalika Ariyaratne, W. M. N. P. Buddhadasa, Sunil Ratnayake, Malani Wickramasinghe

摘要

引言：天气变化影响登革热传播。本研究旨在发现斯里兰卡康提市登革热-气象相关模式，并将所得结果与类似研究进行比较，从而提供更有效的登革热防控方法。

方法：搜集 2003-2012 年康提市登革热报告病例和年中人口数据，计算出周发病率。从两个气象站获得每日天气数据并转换成周数据。应用小波时间序列分析研究登革热发病率和气象变量之间的相关性模式，计算交叉相关系数以发现关联强度。

结果：本研究发现登革热发病率与降雨量、阴雨天数、最低气温、白天夜晚时长及平均值和湿度呈正相关，一般会有 5 至 7 周的滞后。此外，还发现了登革热发病率与最高气温和平均气温、日照时长和风向相关，但有更长的滞后期。登革热和风向呈负相关。

结论：结果表明，当地登革热发病率与降雨量、温度、日照时长和风相关。在全球变暖背景下，我们提出改进登革热管理和控制方法。我们还注意到，登革天气相关性研究结果随着数据分析的变化而变化。

Translated from English version into Chinese by Chen Jin, edited by Yang Pin, through

CHINESE CENTER FOR DISEASE CONTROL AND PREVENTION
NATIONAL INSTITUTE OF PARASITIC DISEASES

Une étude de la corrélation entre la dengue et le climat à Kandy, au Sri Lanka (2003–2012)

N. D. B. Ehelepola, Kusalika Ariyaratne, W. M. N. P. Buddhadasa, Sunil Ratnayake, Malani Wickramasinghe

RÉSUMÉ

Contexte: Les variables climatiques influent sur la transmission de la dengue. Cette étude vise à élaborer un modèle de corrélation entre la dengue et le climat à Kandy, au Sri Lanka, à comparer les résultats avec ceux obtenus lors d'études similaires, et à améliorer les moyens de contrôle et de prévention de la dengue.

Méthode: Nous avons recueilli des données sur les cas de dengue signalés à Kandy et des données sur la population en milieu d'année de 2003 à 2012, puis nous avons calculé les incidences hebdomadaires. Des données climatiques quotidiennes ont été fournies par deux stations météorologiques, et ont été converties en données hebdomadaires. Nous avons examiné les modèles de corrélation entre l'incidence de la dengue et les variables climatiques en utilisant la méthode d'analyse par ondelettes des séries temporelles, et avons ensuite calculé les coefficients d'intercorrélation afin de déterminer les niveaux de corrélation.

Résultats: Nous avons relevé une corrélation positive entre l'incidence de la dengue et la hauteur des précipitations en millimètres, le nombre de jours pluvieux et humides, la température minimale, la nuit et le jour, ainsi que l'humidité moyenne, principalement avec un décalage de cinq à sept semaines. De plus, nous avons constaté l'existence de corrélations entre l'incidence de la dengue et les températures maximales et moyennes, les heures d'ensoleillement et le vent, avec des périodes de décalage plus longues. Une corrélation négative a été observée entre les incidences de dengue et le parcours du vent.

Conclusion: Nos résultats ont montré que les précipitations, la température, l'humidité, les heures d'ensoleillement, et le vent étaient corrélés avec l'incidence locale de dengue. Nous avons proposé des méthodes afin d'améliorer les procédures de gestion de la dengue et de la maîtriser, en ces temps de réchauffement climatique. Nous avons aussi constaté que les résultats des études de corrélation entre la dengue et le climat pouvaient varier selon l'analyse des données.

Translated from English version into French by Myriam Grandchamp, through

TRANSLATORS
WITHOUT BORDERS

Изучение соотношения между заболеваемостью тропической лихорадкой и погодой в Канди, Шри-Ланка (2003-2012 г.г.)

N. D. B. Ehelepola, Kusalika Ariyaratne, W. M. N. P. Buddhadasa, Sunil Ratnayake, Malani Wickramasinghe

КРАТКОЕ ОПИСАНИЕ

Базовая информация: Переменчивая погода оказывает воздействие на распространение вируса тропической лихорадки. Настоящее исследование направлено на то, чтобы выявить схему корреляции между погодой и заболеваемостью тропической лихорадкой в Канди, Шри-Ланка, сравнить полученные результаты с результатами аналогичных исследований и определить способы улучшения контроля и профилактики распространения тропической лихорадки.

Методология: Мы собрали данные о зафиксированных случаях тропической лихорадки в Канди и информацию о среднегодовом населении в период с 2003 по 2012 г.г. и рассчитали число случаев заболевания в неделю. Мы получали ежедневные сведения о погодных условиях от двух метеорологических станций и преобразовывали их в недельные данные. Мы изучили схемы корреляции между числом случаев заболевания тропической лихорадкой и изменениями погодных условий с помощью вейвлет-анализа временных рядов, а затем рассчитали коэффициенты взаимной корреляции для определения мощности таких корреляций.

Результаты: Мы обнаружили положительную корреляцию между числом случаев заболевания тропической лихорадкой и количеством выпавших осадков в миллиметрах, числом дождливых и сырых дней, минимальной температурой, количеством дневных и ночных часов, а также средним уровнем влажности в период в 5-7 недель. Кроме того, мы выявили корреляцию между числом случаев заболевания тропической лихорадкой и максимальными и средними температурами, количеством солнечных часов и наличием ветра в течение более долгих периодов. Число случаев заболевания тропической лихорадкой оказалось в отношениях отрицательной корреляции с количество ветреных дней.

Вывод: Согласно полученным результатам, количество выпавших осадков, температура, влажность, количество солнечных часов и наличие ветра связаны с числом случаев заболевания тропической лихорадкой в данной местности. Мы предложили способы усовершенствования порядка контроля распространения тропической лихорадки в период всеобщего глобального потепления. Кроме того, мы отметили, что результаты исследований соотношения между погодными условиями и случаями заболевания тропической лихорадкой могут варьироваться в зависимости от анализа данных.

Translated from English version into Russian by Irina Zayonchkovskaya, through

TRANSLATORS WITHOUT BORDERS

Un estudio sobre la correlación entre el dengue y el tiempo en Kandy, Sri Lanka (2003-2012)

N. D. B. Ehelepola, Kusalika Ariyaratne, W. M. N. P. Buddhadasa, Sunil Ratnayake, Malani Wickramasinghe

RESUMEN

Antecedentes: Las variables en el tiempo afectan la transmisión del dengue. Este estudio tenía como objetivo identificar un patrón de correlación entre dengue y tiempo en Kandy, Sri Lanka, comparar los resultados con aquellos de estudios similares, y establecer métodos para un mejor control y prevención del dengue.

Método: Recolectamos información de casos de dengue reportados en Kandy e información de la población a mitad de año entre 2003 y 2012, y calculamos las incidencias semanales. Obtuvimos información del tiempo diario

de dos estaciones meteorológicas y las convertimos a información semanal. Estudiamos los patrones de correlación entre la incidencia de dengue y las variables de tiempo utilizando un análisis ondicular de series de tiempo, y luego calculamos los coeficientes de correlación cruzada para encontrar la magnitud de las correlaciones.
Resultados: Encontramos una correlación positiva entre la incidencia de dengue y la precipitación en milímetros, la cantidad de días lluviosos y húmedos, la temperatura mínima y el día y la noche, al igual que el promedio, humedad, por lo general con un retraso de cinco a siete semanas. Adicionalmente, encontramos una correlación entre la incidencia de dengue y las temperaturas máximas y promedio, horas de sol y viento, con mayores períodos de retraso. Las incidencias de dengue mostraron una correlación negativa con el recorrido del viento.
Conclusión: Nuestros resultados mostraron que las precipitaciones, temperatura, humedad, horas de sol y el viento tienen una correlación con la incidencia local del dengue. Hemos sugeridos métodos para mejorar las rutinas de manejo del dengue y para su control en esta era del calentamiento global. También notamos que los resultados de los estudios de correlación dengue-tiempo pueden variar dependiendo del análisis de la información.

Translated from English version into Spanish by Maria Alejandra Aguada, through

TRANSLATORS
WITHOUT BORDERS
